# Mortality of Japanese patients with Leigh syndrome: Effects of age at onset and genetic diagnosis

**DOI:** 10.1002/jimd.12218

**Published:** 2020-02-10

**Authors:** Erika Ogawa, Takuya Fushimi, Minako Ogawa‐Tominaga, Masaru Shimura, Makiko Tajika, Keiko Ichimoto, Ayako Matsunaga, Tomoko Tsuruoka, Mika Ishige, Tatsuo Fuchigami, Taro Yamazaki, Yoshihito Kishita, Masakazu Kohda, Atsuko Imai‐Okazaki, Yasushi Okazaki, Ichiro Morioka, Akira Ohtake, Kei Murayama

**Affiliations:** ^1^ Department of Metabolism Chiba Children's Hospital Chiba Japan; ^2^ Department of Pediatrics and Child Health Nihon University School of Medicine Tokyo Japan; ^3^ Department of Pediatrics Saitama Medical University Saitama Japan; ^4^ Intractable Disease Research Center Graduate School of Medicine, Juntendo University Tokyo Japan; ^5^ Department of Clinical Genomics Saitama Medical University Saitama Japan

**Keywords:** early onset, genetic diagnosis, Japanese patients, Leigh syndrome, mortality

## Abstract

Leigh syndrome is a major phenotype of mitochondrial diseases in children. With new therapeutic options being proposed, assessing the mortality and clinical condition of Leigh syndrome patients is crucial for evaluating therapeutics. As data are scarce in Japan, we analysed the mortality rate and clinical condition of Japanese Leigh syndrome patients that we diagnosed since 2007. Data from 166 Japanese patients diagnosed with Leigh syndrome from 2007 to 2017 were reviewed. Patients' present status, method of ventilation and feeding, and degree of disability as of April 2018 was analysed. Overall, 124 (74.7%) were living, 40 (24.1%) were deceased, and 2 (1.2%) were lost to follow‐up. Median age of living patients was 8 years (1‐39 years). Median length of disease course was 91 months for living patients and 23.5 months for deceased patients. Nearly 90% of deaths occurred by age 6. Mortality rate of patients with onset before 6 months of age was significantly higher than that of onset after 6 months. All patients with neonatal onset were either deceased or bedridden. *MT‐ATP6* deficiency caused by m.8993T>G mutation and *MT‐ND5* deficiency induced a severe form of Leigh syndrome. Patients with *NDUFAF6*, *ECHS1*, and *SURF1* deficiency had relatively mild symptoms and better survival. The impact of onset age on prognosis varied across the genetic diagnoses. The clinical condition of many patients was poor; however, few did not require mechanical ventilation or tube‐feeding and were not physically dependent. Early disease onset and genetic diagnosis may have prognostic value.

## 1 INTRODUCTION

Leigh syndrome (LS; OMIM 256000) is a progressive neurodegenerative disease, mainly affecting children, associated with dysfunction of mitochondrial oxidative phosphorylation.^1^ It was originally defined neuropathologically by bilateral necrotic lesions in the basal ganglia and/or brain stem.^2^ Psychomotor regression or retardation and symptoms related to brainstem dysfunction are the major clinical manifestations of LS.^3^ Onset is often in infancy or early childhood, and many patients face early deaths^4^; however, information on the actual mortality rate of LS patients or the long‐term severity of their disability is scarce. With deeper understanding of mitochondrial diseases, new therapeutic options are being proposed and clinical trials are in process.^5^, ^6^ To monitor the effect of new treatments, basic data on mortality and clinical conditions of LS patients is essential.

We have previously reported the biochemical and genetic backgrounds of 106 cases of LS and Leigh‐like syndrome.^7^ With an additional 60 patients diagnosed since our previous report, we reviewed the present status of these patients as of April 2018 to analyse the mortality rate, clinical conditions, and risk factors that affect the outcome of LS patients in Japan.

## 2 MATERIALS AND METHODS

One hundred and sixty‐six patients diagnosed with LS or Leigh‐like syndrome were analysed. Chiba Children's Hospital, Saitama Medical University, and Juntedo University received approval for comprehensive study of mitochondrial disease including biochemical and genetic analysis from their appropriate ethics review boards. Written informed consent was obtained from the parents of each patient at referral. Patients who fulfilled the stringent criteria of LS, as defined by Rahman et al,^8^ were diagnosed with LS. Patients recognised as Leigh‐like by Rahman's definition (patients with atypical or normal neuroimaging results or those with typical neuroimaging results but normal lactate levels in the serum and cerebrospinal fluid^8^) were also included in this study. Patients underwent enzymatic analysis by spectrophotometric assays for complex I, II, III, and IV using skin fibroblasts and/or skeletal muscle biopsy samples. Patients with mitochondrial respiratory chain defects by enzyme assay were analysed for mitochondrial deoxyribonucleic acid (mtDNA) mutations by whole mtDNA sequencing. Where no causative mtDNA mutations were found, whole‐exome sequencing with next‐generation sequencing for nuclear DNA (nDNA) mutations was performed. Those with negative enzyme assay results were screened for mutations using a targeted gene panel of 251 nuclear genes known to cause mitochondrial diseases as well as using the whole mitochondrial genome. Detailed information on the diagnostic procedure is available in our previous reports.^7^, ^9^ We reviewed the present status of each patient as of April 2018. Information on need for mechanical ventilation and tube‐feeding, and physical dependency was also analysed for living patients. We also made inquiry via the Japan Mitochondrial Disease Research Organization Data Bank (J‐MO Bank), a Japanese patient registry for mitochondrial disorders, and the parents of some patients registered in J‐MO Bank responded directly to our inquiry.

Statistical analysis was performed using Microsoft Excel 2016® (Microsoft, Richmond, Washington). The χ^2^ test for categorical variables and unpaired Student's *t* test for continuous variables were used to compare between two groups. Kaplan‐Meier (KM) survival analyses were used to evaluate the survival outcome. KM plots were generated using R software version 1.1.423 (The R Foundation for Statistical Computing [http://www.r-project.org/]) and “survminer” package version 0.4.6 (https://cran.r-project.org/web/packages/survminer/index.html). The survival rates between two groups were compared using a log‐rank test. All statistical tests were two‐sided and a *P* value of <.05 was considered statistically significant.

## 3 RESULTS

One hundred and sixty‐six patients were surveyed. One hundred and twenty‐three patients were diagnosed LS and 43 were diagnosed Leigh‐like syndrome. Those diagnosed with LS all showed typical bilateral necrotic lesions in the basal ganglia with magnetic resonance imaging (MRI) at some point during the disease course. Those diagnosed Leigh‐like syndrome lacked typical MRI findings or high levels of lactate or the lactate to pyruvate ratio in either serum or cerebral spinal fluid. Forty‐nine patients had been diagnosed with nDNA mutations (29.5%), and 54 with mtDNA mutations (32.5%). The list of mutated genes is provided in Table 1, and specific mutations are presented in Table S1. Most of the causative genes were primarily linked to mitochondrial oxidative phosphorylation (OXPHOS), but there were also genes known to cause secondary OXPHOS defects.^10^ Enzymatic analysis revealed respiratory chain complex deficiency in 98 patients with isolated complex I deficiency in 49 patients, isolated complex IV deficiency in 21 patients, and compound complex deficiency in 25 patients. There were 55 patients with no enzymatic defect. The remaining 13 patients had not performed enzymatic analysis.

**Table 1:** Genetic diagnosis of Leigh syndrome and Leigh‐like syndrome patients

Specific to OXPHOS biogenesis
OXPHOS subunits
MT‐ND1(4)^a^, MT‐ND3(9), MT‐ND4, MT‐ND5(7), MT‐ND6(11), MT‐ATP6(18)
NDUFA1, NDUFS4, NDUFS6, NDUFS7, NDUFV2, UQCRC2
OXPHOS assembly factors
NDUFAF6(8), COX15, SCO2(2), SURF1(7)
Electron carriers
COQ4
mtRNA expression/processing
HSD17B10(2)
mtDNA biogenesis/aminoacylation
MT‐TE, MT‐TL1(2), MT‐TW, GTPBP3, NARS2(2)
Secondary OXPHOS defect
BOLA3(2), SLC19A3(2), PDHA1(3), ECHS1(10), BCAP31, DNML1

a Numbers in parentheses show number of patients.

**Figure 1:**
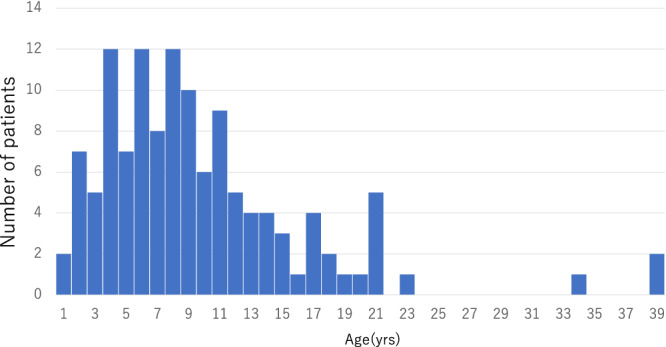
Age distribution of living patients (N = 124)

**Figure 2:**
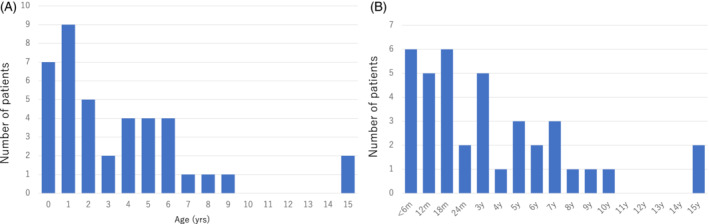
A, Distribution of age at death (N = 40). B, Length of disease course (deceased cases; N = 38)

One hundred and twenty‐four patients were alive in April of 2018 (74.7%). Forty patients were deceased (24.1%), and two were lost to follow‐up (1.2%). Of the 40 deceased patients, 11 patients were deceased at diagnosis, and 29 patients had died after the diagnosis. The median age of the living patients was 8 years (range, 1‐39 years; Figure 1) with a median length of disease course of 91 months (data not shown). The median age of death was 31.5 months with the most deaths (9 out of 40) occurring at 1 year of age; 35 patients (87.5%) died by the age of 6 years (Figure 2). The median length of disease course for the deceased was 23.5 months (Figure 2).

**Figure 3:**
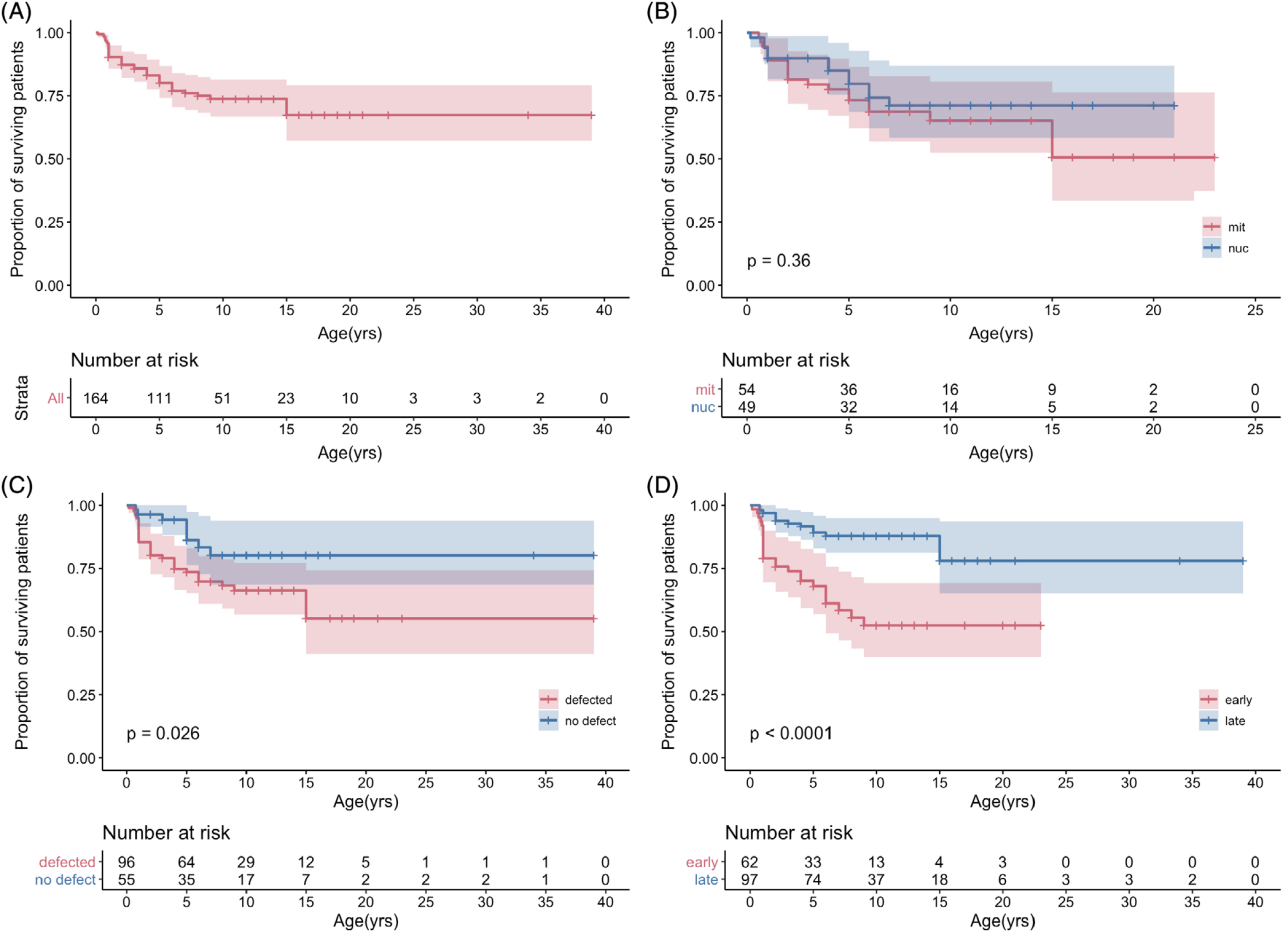
A, Overall survival rate with number of subjects at risk. B, Survival rate by types of mutation with number of subjects at risk. In red, patients with mutations in mitochondrial DNA. In blue, patients with mutations in nuclear DNA. They showed similar mortality (*P* = .36). mt: mutation in mitochondrial DNA. nuc: mutation in nuclear DNA. C, Survival rate by complex deficiency with number of subjects at risk. In red, patients with defected enzyme activities. In blue, patients with no defect detected. Patients with no defect detected showed a better survival rate compared to patients with defected enzyme activities (*P* = .026). D, Survival rate by age of onset with number of subjects at risk. In red, patients with disease presentation before 6 months of age (early onset). In blue, patients with disease presentation in and after 6 months of age (late onset). Early onset showed significantly higher mortality (*P* < .0001). Tick marks show censored cases. Shaded areas show 95% confidence interval

The overall survival curve is shown in Figure 3. There was no statistical difference between the mortality rates of LS and Leigh‐like syndrome patients (Figure S1). Patients with a genetic diagnosis had higher mortality compared to patients without a genetic diagnosis (Figure S2). Patients with mtDNA mutation had similar mortality against those with nuclear DNA mutations (Figure 3). Patients with enzymatic defects had higher mortality compared to patients with normal complex activity (Figure 3). There was no significant difference between complex I deficiency and complex IV deficiency. Those with compound complex deficiency had the lowest survival, but there was no statistical difference (Figure S3). The survival analysis according to the age of onset of the disease is shown in Figure 3. Mortality was significantly higher for patients with disease onset before 6 months of age (early onset; *P* < .0001). At the time of the data analysis, 40.3% of the 62 patients in the early onset group had died, whereas only 14.3% of the 98 patients with onset after 6 months of age (late onset) had died (*P* < .0001). All the patients with neonatal onset were either deceased or bedridden.

**Figure 4:**
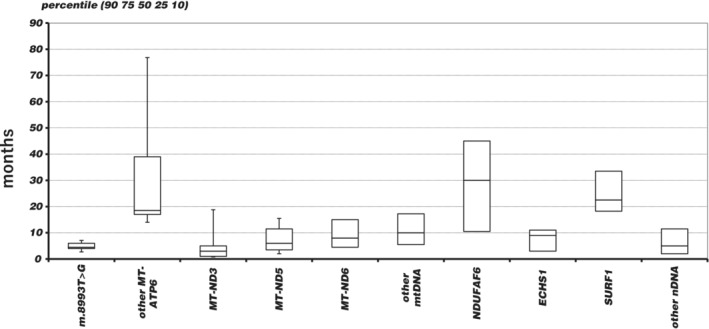
Distribution of age at onset by genetic diagnosis

**Figure 5:**
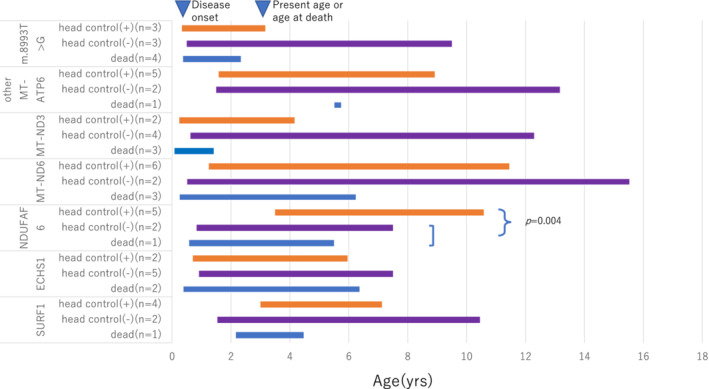
Length of disease course by genetic diagnosis and severity. There was a significant difference in onset age between *NDUFAF6* deficient patients who maintained head control (N = 5) and patients either deceased or who had lost head control (N = 3; *P* = .004). The left end of each bar shows the median age of disease onset. The right end of each bar shows the present age or age at death. Head control (+): alive with head control (+). Head control (−): alive but head control lost or never acquired

The clinical conditions of living patients were variable. Twenty‐five patients depended on invasive mechanical ventilation, while three used noninvasive positive pressure ventilation devices only. Three more had tracheostomy for airway purposes. Fifty‐eight patients (46.8%) were tube‐fed. As for physical disability, 61 patients had lost or never acquired head control, 13 patients had maintained head control but could not move by themselves and were basically bedridden, 30 patients were either able to roll‐over, sit, or stand with holding, and 20 patients were able to walk. Outcome according to the major responsible genes is summarised in Table 2. Patients with some type of mtDNA mutations showed high mortality. For example, only one out of 7 patients with any mutation of *MT‐ND5* had survived, and 4 out of 10 patients with an m.8993T>G mutation of *MT‐ATP6* were deceased. Patients with other mutations of the same *MT‐ATP6* exhibited a milder course of the disease. Patients with mutations in *NDUFAF6*, *SURF1*, and *ECHS1* had better outcomes than those with mutations in other nDNA encoded genes. Distribution of onset age in months according to causative genes is compared in Figure 4. Patients with mutations in *MT‐ND3* and patients with the m.8993T>G mutation had the earliest onset. *ECHS1* deficiency had relatively early onset among nDNA group. Patients with other mutations in *MT‐ATP6* or deficiency in *NDUFAF6* or *SURF1* showed later disease onset age with a wider distribution. Among patients with a genetic diagnosis that showed a narrow distribution of onset age, those who died and those who are still living could not be distinguished by the onset age. On the contrary, for patients with *NDUFAF6* deficiency, later onset was related with better outcome. The mean onset age of *NDUFAF6* patients living with head control intact (N = 5) was 42.2 months compared to 9 months of patients either deceased or who had lost head control (N = 3; *P* = .004; Figure 5).

**Table 2:** Survival status and clinical conditions of patients with major genetic diagnosis

Gene	N	Alive	Clinical condition of living patients			
			Mechanical ventilation	Tube feeding	Head control lost/never achieved	Bedridden
nDNA						
*NDUFAF6*	8	7	0	1	2	2
*ECHS1*	10	8	1	5	6	8
*SURF1*	7	6	3	2	2	2
mtDNA						
*MT‐ATP6*						
m.8993T>G	10	6	2	3	3	4
other MT‐ATP6	8	7	0	0	2	2
*MT‐ND3*	9	6	5	5	4	6
*MT‐ND5*	7	1	0	0	0	0
*MT‐ND6*	11	8	1	4	2	4

## 4 DISCUSSION

This study was undertaken to review data on survival and the clinical condition of Japanese LS and Leigh‐like syndrome patients. We observed that of the 166 patients, there were 124 (74.7%) living patients, 40 (24.1%) deceased patients, and 2 (1.2%) patients lost to follow‐up. The mortality rate of patients with disease onset before 6 months of age was significantly higher than that of patients with onset after 6 months. Genetic diagnosis was another factor associated with poor prognosis.

The mortality rate of LS patients is reported to be generally high. Rahman et al reported a survival rate of 20% by the age of 20 years.^8^ Lee reported that 57% of 14 patients had died by the age of 1 year and 6 months.^11^ Naess et al reported a high mortality rate of 68% by the age of 15 years in patients with mitochondrial DNA mutations.^12^ Sofou et al analysed the disease course of 130 LS patients in Europe and reported a better survival rate of about 60% by the age of 10 years and 40% by the age of 20 years.^13^ However, there is no data on the mortality rate of LS or Leigh‐like syndrome patients in Asia. The present study reports the mortality data of Japanese patients with LS and Leigh‐like syndrome for the first time. The data shows that not all patients suffer from early deaths. While half of the deceased patients had died during the first 2 years after onset, many patients have survived beyond the initial crisis, and the median length of disease course of living patients was 91 months. Survival rate by the age of 15 years estimated by KM analysis is 70% in our cohort (Figure 3), which is considerably higher than the survival rate reported from other countries. This observation can be attributed to several reasons. Older children with the disease who were referred to our study had already survived through the first few critical years, and therefore, our cohort may have been biased towards better survival. However, those patients did not constitute a large part of our cohort (25 patients were older than 7 years of age at referral), and the influence should be limited. Increasing awareness of mitochondrial disease among the Japanese paediatric community may have contributed to the better survival, with more children being referred and diagnosed with the disease before an acute onset. Another reason for the apparently better survival in our cohort compared to that in other parts of the world may be attributed to the Japanese paediatric practice, which does not withdraw life support devices such as the ventilator once it is started.

Early onset of the disease and genetic diagnosis had an influence on the prognosis. Onset before 6 months of age has been reported to be an important prognostic factor.^12^, ^13^ Our report adds further strong evidence to this observation. Neonatal onset had a great impact on outcomes, and all patients with neonatal onset were reported to be either deceased or bedridden. Onset from 1 to 5 months of age was also associated with extreme severity, with 80% of patients deceased or bedridden.

Although deficiency in the respiratory chain complex is an important biochemical finding in diagnosing LS, there has been no clear relation reported in literature between outcome and biochemical defects. In our cohort, patients exhibiting enzymatic defects had a higher mortality rate compared to patients with no enzymatic defects with a *P*‐value of .0260. Defects in complex I have been associated with severe multisystem organ involvement and a poor outcome.^14^ However, isolated complex I deficiency did not show a significantly higher mortality rate compared to other complex or compound complex deficiency in our study (Figure S3). Poor prognosis of compound complex deficiencies such as deficiencies of complex I + IV or of complex I + III + IV may imply that they are detected in a more advanced stage of the disease.

LS caused by mtDNA mutations showed a similar mortality rate compared to those caused by nDNA mutations in our cohort. Naess et al have reported severe outcomes in patients with mtDNA mutated LS.^12^ The high mortality rate of mtDNA mutated cases was attributed to the high mortality rates of patients with mutations in *MT‐ND5* (85.7%) and m.8993T>G mutation of *MT‐ATP6* (40%). The mortality rate of nDNA mutation cases was lower because the major causative genes, namely, *NDUFAF6*, *ECHS1*, and *SURF1*, constituting a half of the nDNA mutation cases in our cohort, took a milder disease course. LS with *SURF1* mutation has been reported to be associated with longer survival than other types of LS,^13^, ^15^, ^16^ and our report adds further strong evidence for this. There is an increasing number of cases with *ECHS1* deficiency presenting with LS, and the severity of the disease is variable.^17^, ^18^, ^19^, ^20^ Patients in our cohort may have mutations that result in a milder course than those reported from other countries. Favourable outcomes with *NDUFAF6* deficiency were reported in a recent study by Baide‐Mairena et al.^21^ LS is genetically heterogeneous and is caused by defects in many different molecules involved in OXPHOS.^22^ Our observations highlight the importance of identifying the biochemical and genetic background of the disease in each case to discuss the mortality due to the disease.

The clinical condition of many patients was very severe; nonetheless, we also observed patients who did not require mechanical ventilation, who were on oral feeding, and who maintained the ability to walk. There was no apparent genotype‐phenotype correlation. We did not explicitly enquire for resolution of symptoms, but since 31% of patients in the previous report presented with respiratory distress at referral,^7^ we presume that some patients may have recovered from their original condition.

This study has some limitations. The possibility of bias towards a favourable outcome has been discussed above. We also did not collect detailed information on the condition of the patients. For patients with diseases that impose severe disabilities such as LS, subtle changes in the patients' condition may make a large difference in the patients' quality of life and to the burden on caregivers. To monitor the effects of new treatments, more detailed information on the clinical condition of the patients may be necessary. This data may be best collected via a patient registry system. Meanwhile, our study is the first report on the mortality rate and the clinical conditions of patients with LS in Japan. We believe that the present study provides indispensable knowledge regarding the outcome and present status of these Japanese LS patients.

## CONFLICT OF INTEREST

Erika Ogawa, Takuya Fushimi, Minako Ogawa‐Tominaga, Masaru Shimura, Makiko Tajika, Keiko Ichimoto, Ayako Matsunaga, Tomoko Tsuruoka, Mika Ishige, Tatsuo Fuchigami, Taro Yamazaki, Yoshihito Kishita, Masakazu Kohda, Atsuko Imai‐Okazaki, Yasushi Okazaki, Ichiro Morioka, Akira Ohtake, and Kei Murayama declare that they have no conflict of interest.

## AUTHOR CONTRIBUTIONS

E.O. designed and drafted the manuscript. T.F., M.To., M.S., M.Ta., K.I., A.M., T.T., and T.Y. collected, analysed, and interpreted the data. M.I., T.F., and I.M. read and revised the manuscript critically for important intellectual content. Y.K. and M.K. managed the genetic analysis. A.I.‐O. provided technical support on statistical analyses. Y.O., A.O., and K.M. planned the whole research project. K.M. supervised the conception and preparation of the manuscript.

## COMPLIANCE WITH ETHICAL STANDARDS

All procedures followed were in accordance with the ethical standards of the responsible committee on human experimentation (institutional and national) and with the Helsinki Declaration of 1975, as revised in 2000 (5). Chiba Children's Hospital, Saitama Medical University, and Juntedo University received approval for comprehensive study of mitochondrial disease including biochemical and genetic analysis from their appropriate ethics review boards.

## PATIENT CONSENT STATEMENT

Informed consent was obtained from the parent(s) of each patient for being included in the study.

## Supporting information


**Table S1** List of mutation.

**Figure S1** Survival rate by phenotypes with number of subjects at risk. In red, Leigh syndrome patients. In blue, Leigh‐like patients. There was no statistical difference between the mortality rate of Leigh syndrome patients and Leigh‐like patients (*P* = 0.34). Tick marks show censored cases. Shaded areas show 95% confidence interval. LS: Leigh syndrome. LL: Leigh‐like syndrome.

**Figure S2** Survival rate by genetic diagnosis with number of subjects at risk. In red, patients with a genetic diagnosis. In blue, patients without a genetic diagnosis. Patients with a genetic diagnosis had a higher mortality compared to patients without (*P* = 0.034). Tick marks show censored cases. Shaded areas show 95% confidence interval.

**Figure S3** Survival rate by types of complex deficiency with number of subjects at risk. Patients with a complex I deficiency had a similar mortality with patients with complex IV deficiency or compound complex deficiency (*P* = 0.071). Tick marks show censored cases.
