# Supplementary figures and images for: Mortality of Japanese patients with Leigh syndrome: Effects of age at onset and genetic diagnosis
Source: J Inherit Metab Dis. 2020 Feb 10;43(4):819–26. doi: 10.1002/jimd.12218 (PMC7383885; doi:10.1002/jimd.12218)

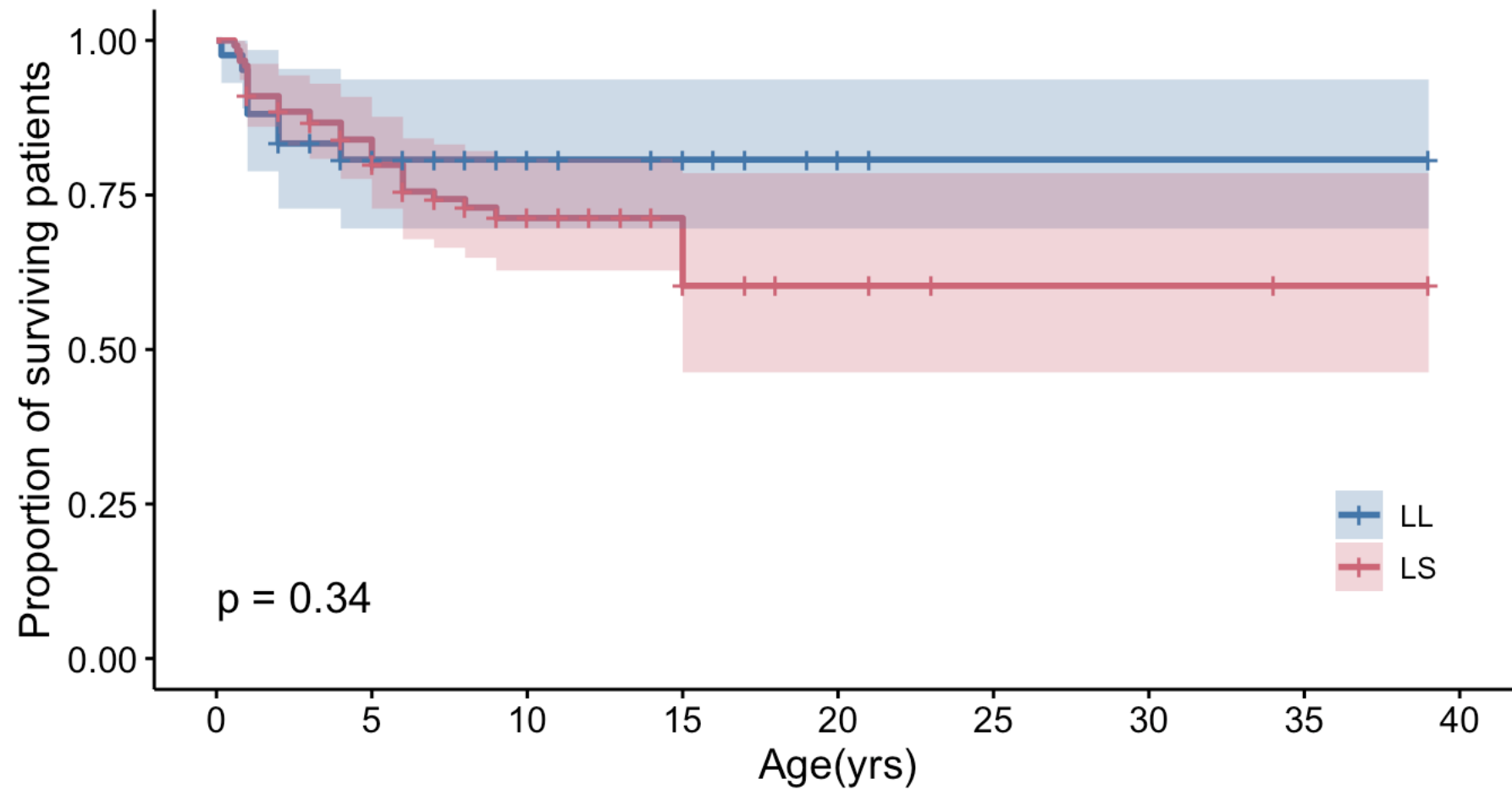

Number at risk

|    |     |    |    |    |    |    |    |    |    |
|----|-----|----|----|----|----|----|----|----|----|
| LL | 42  | 29 | 16 | 10 | 5  | 1  | 1  | 1  | 0  |
| LS | 122 | 82 | 35 | 13 | 5  | 2  | 2  | 1  | 0  |
|    | 0   | 5  | 10 | 15 | 20 | 25 | 30 | 35 | 40 |

Age(yrs)

Supplement: Supplementary file 2 — Figure S1 Survival rate by phenotypes with number of subjects at risk. In red, Leigh syndrome patients. In blue, Leigh‐like patients. There was no statistical difference between the mortality rate of Leigh syndrome patients and Leigh‐like patients (P = 0.34). Tick marks show censored cases. Shaded areas show 95% confidence interval. LS: Leigh syndrome. LL: Leigh‐like syndrome. [file JIMD-43-819-s001.pdf]
