# Supplementary material for: Mortality of Japanese patients with Leigh syndrome: Effects of age at onset and genetic diagnosis
Source: J Inherit Metab Dis. 2020 Feb 10;43(4):819–26. doi: 10.1002/jimd.12218 (PMC7383885; doi:10.1002/jimd.12218)
Supplement: Supplementary file 3 — Figure S2 Survival rate by genetic diagnosis with number of subjects at risk. In red, patients with a genetic diagnosis. In blue, patients without a genetic diagnosis. Patients with a genetic diagnosis had a higher mortality compared to patients without (P = 0.034). Tick marks show censored cases. Shaded areas show 95% confidence interval. [file JIMD-43-819-s002.pdf]

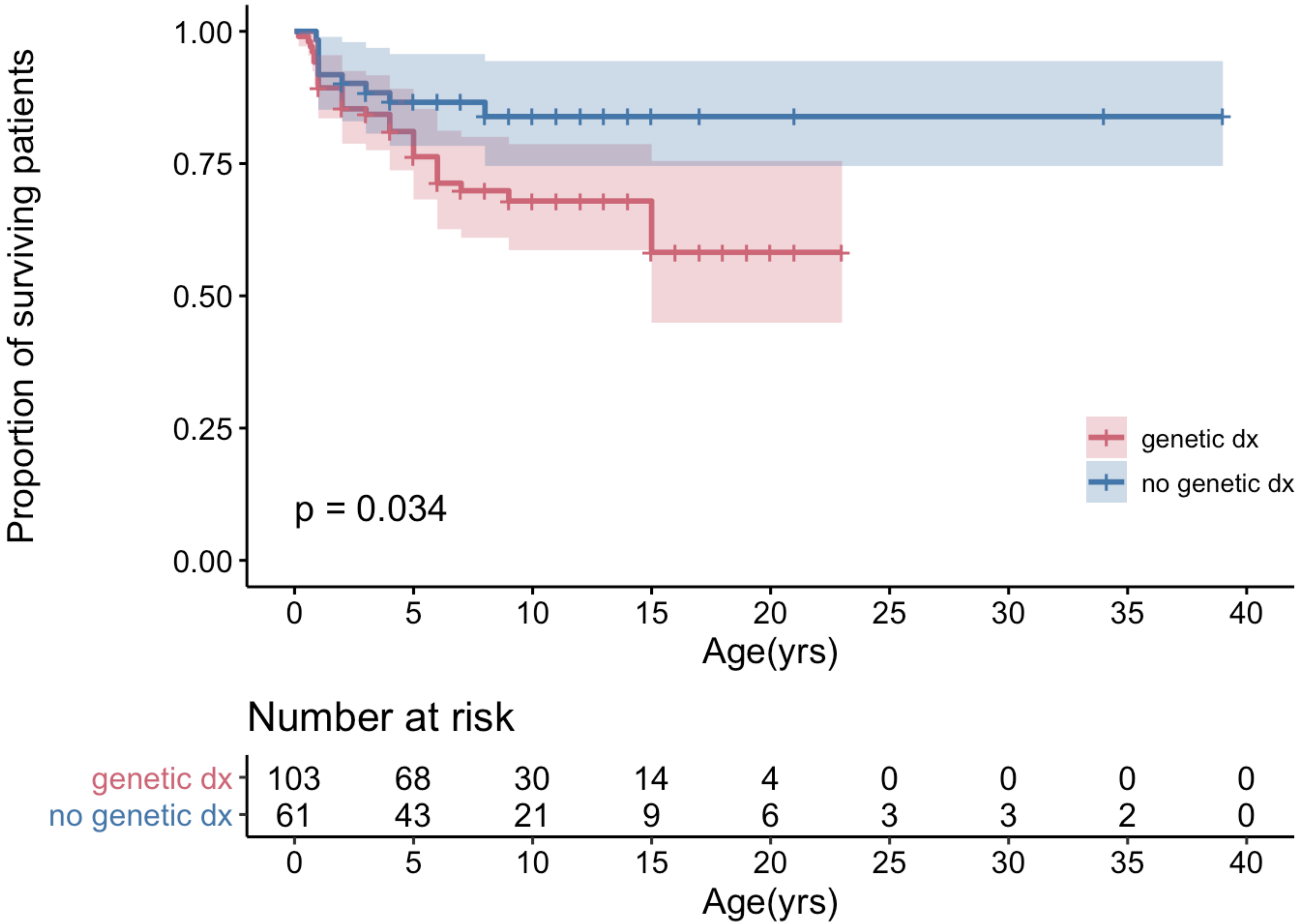

Proportion of surviving patients
1.00
0.75
0.50
0.25
0.00
p = 0.034
genetic dx
no genetic dx
0
5
10
15
20
25
30
35
40
Age(yrs)
Number at risk
genetic dx 103 68 30 14 4 0 0 0 0
no genetic dx 61 43 21 9 6 3 3 2 0
0
5
10
15
20
25
30
35
40
Age(yrs)
