# Supplementary material for: Mortality of Japanese patients with Leigh syndrome: Effects of age at onset and genetic diagnosis
Source: J Inherit Metab Dis. 2020 Feb 10;43(4):819–26. doi: 10.1002/jimd.12218 (PMC7383885; doi:10.1002/jimd.12218)
Supplement: Supplementary file 4 — Figure S3 Survival rate by types of complex deficiency with number of subjects at risk. Patients with a complex I deficiency had a similar mortality with patients with complex IV deficiency or compound complex deficiency (P = 0.071). Tick marks show censored cases. [file JIMD-43-819-s003.pdf]

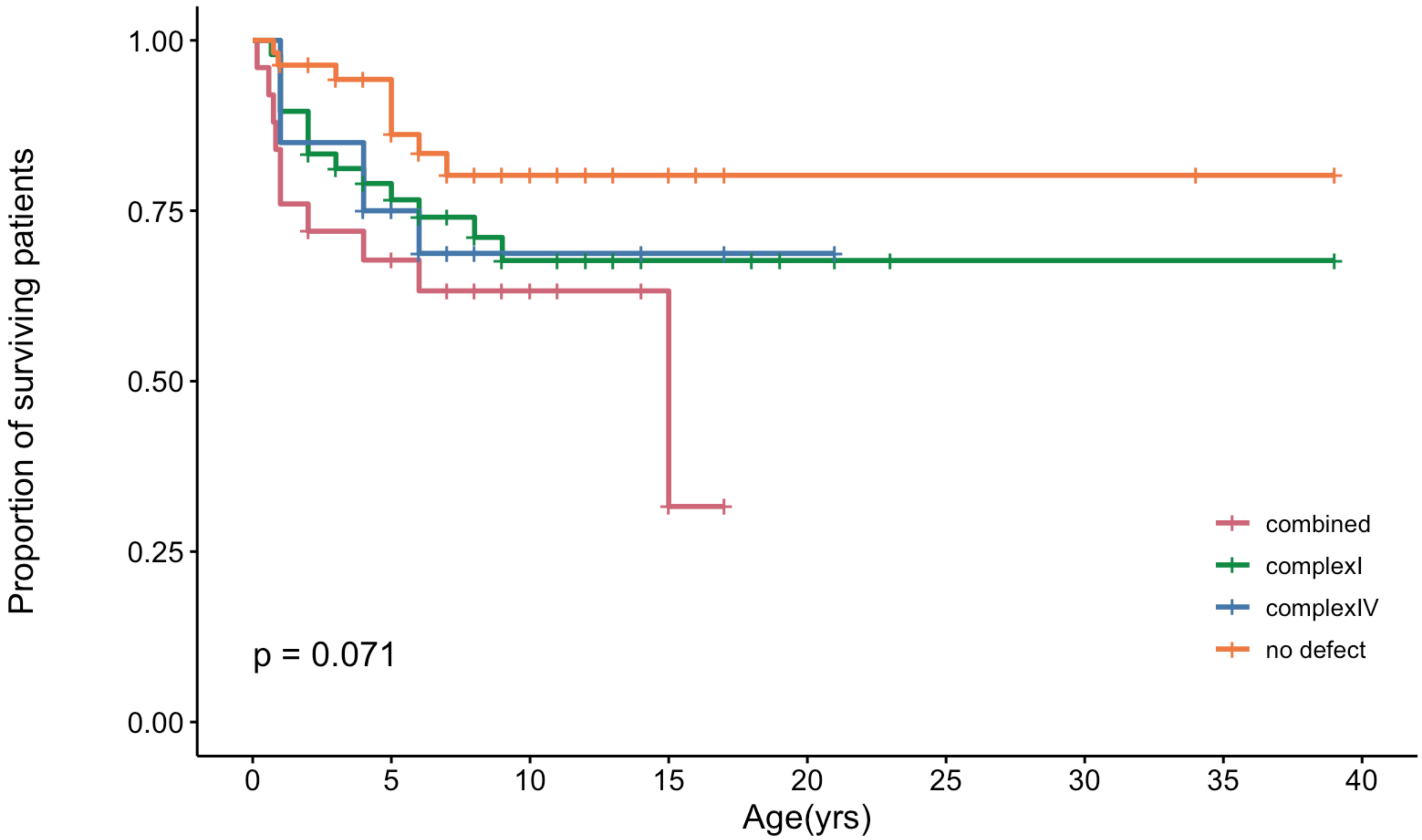

Number at risk

	0	5	10	15	20	25	30	35	40
combined	25	16	7	4	0	0	0	0	0
complexI	48	33	17	5	3	1	1	1	0
complexIV	20	14	5	3	2	0	0	0	0
no defect	55	35	17	7	2	2	2	1	0

Age(yrs)
